# Anamalcules in Our Hydrant Water

**Published:** 1833

**Authors:** 


### V. Animalcules in our Hydrant Water.

Some time after the Epidemic reappeared in our city this spring, it was discovered that our hydrant water, pumped up from the river, abounded in animalcules, many of which could be seen by the naked eye, when examined under favorable circumstances. The phenomenon, of course, excited the 'wonderment' of the 'good people,' and the advocates for the animalcular origin of the pestilence, were fully confirmed. The 'new comers,' as they were considered, were examined by our friends Mr. Hentz, a skilful entomologist, and Mr. Dorfeuille of the

Western Museum, who ascertained that they were microscopic *crustaceæ*—aquatic insects of the great natural order of crabs and lobsters, and about as deleterious, it may be presumed, as those which are served up on the tables of our *gourmands.* After a few days they disappeared. It is said, that a few fish in the reservoir from which the city is supplied, would effectually prevent the reappearance of these aquatic lilliputians.

